# Rectal Gastrointestinal Stromal Tumors

**DOI:** 10.7759/cureus.36361

**Published:** 2023-03-19

**Authors:** Shahed A Dawara, Sameera Naureen, Tasnim R Keloth

**Affiliations:** 1 General Practice, Dubai Health Authority, Dubai, ARE; 2 General Surgery, Dubai Hospital, Dubai, ARE; 3 Histopathology, Dubai Hospital, Dubai, ARE

**Keywords:** neoadjuvant, tyrosine kinase inhibitor (tki), imatinib, gastrointestinal stromal tumors, rectal

## Abstract

Rectal gastrointestinal stromal tumors (GISTs) are rare types of tumors, but the incidence is increasing, and we now know more about the pathogenesis and management of rectal GIST. The main goal is to resect the tumor with negative microscopic margins. With the development of neoadjuvant Imatinib therapy, preoperative reduction in tumor size has become possible, thus introducing the chance for anus-preserving surgery, with better quality of life. We present a case of a 55-year-old female who presented to the emergency department with complaints of bleeding per rectum, abdominal pain, and pain on defecation. A 6-cm mass was detected during the rectal examination and a biopsy confirmed the diagnosis of GIST. The patient was given neoadjuvant imatinib chemotherapy for four months followed by trans-anal resection of the mass. The procedure was done successfully, and she received further adjuvant imatinib for a course of three years. Follow-up by magnetic resonance imaging and a colonoscopy after two years showed no recurrence. The patient is living healthily and doing well.

## Introduction

Gastrointestinal stromal tumors (GISTs) are mesenchymal tumors of the gastrointestinal system, which, in most cases, are KIT (CD117) positive. In the past, these tumors were considered to originate from the smooth muscles of the gastrointestinal tract (GIT), now it is well known that they arise from the interstitial cells of Cajal. The pathogenesis in 80% of the cases is mutations in KIT or PDGFRA receptor tyrosine kinase proteins, which lead to a disruption in their function, resulting in ligand-independent dimerization and activation. Eventually, this activation will rise the cellular proliferation rate and reduce the apoptosis leading to neoplasia. This type of tumor can arise anywhere in the GIT from the esophagus to the anus, including the omentum and mesentery. The stomach is the most common location, and the anorectal GIST is the rarest. In fact, it comprises only 0.1% of all rectal tumors [1]. The overall incidence of GIST is reported to be only 0.68 per 100,000 per year [2]. The first presentation of the disease is commonly a GI bleed; other presentations include acute abdomen due to rupture of the tumor or due to intestinal obstruction. In some cases, it might be an incidental finding. In this case report, we present a case of rectal GIST.

## Case presentation

**Figure 1:**
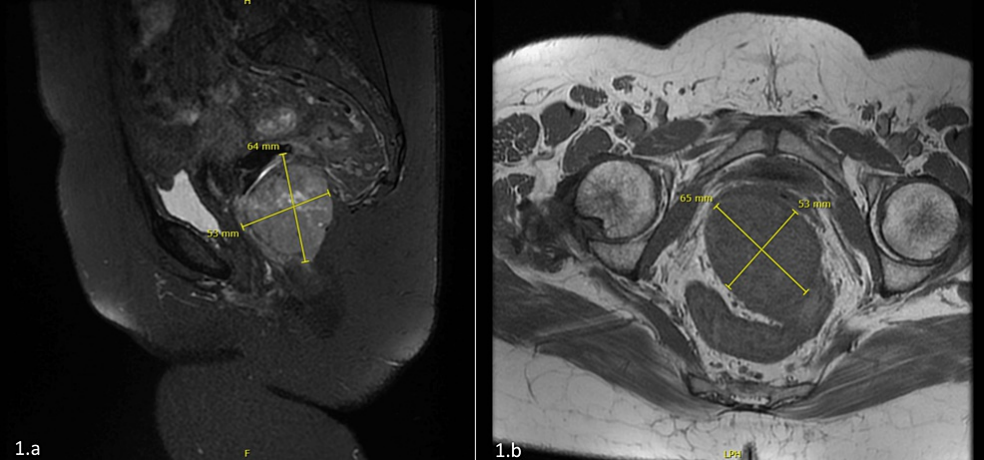
MRI view of the pelvis a: magnetic resonance imaging axial view of the pelvis showing a low rectal mass measuring 6.4 by 5.3 cm. b: magnetic resonance imaging sagittal view of the pelvis

**Figure 2:**
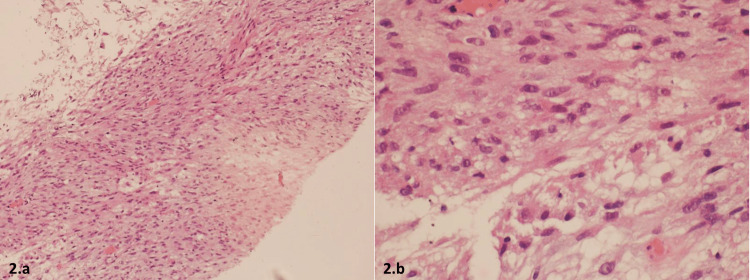
Tru-Cut biopsy a: typical spindle cell of a gastrointestinal tumor. b: high-power view of spindled tumor cells with mitotic figures, mitotic rate 5 per 50 high-power field

**Figure 3:**
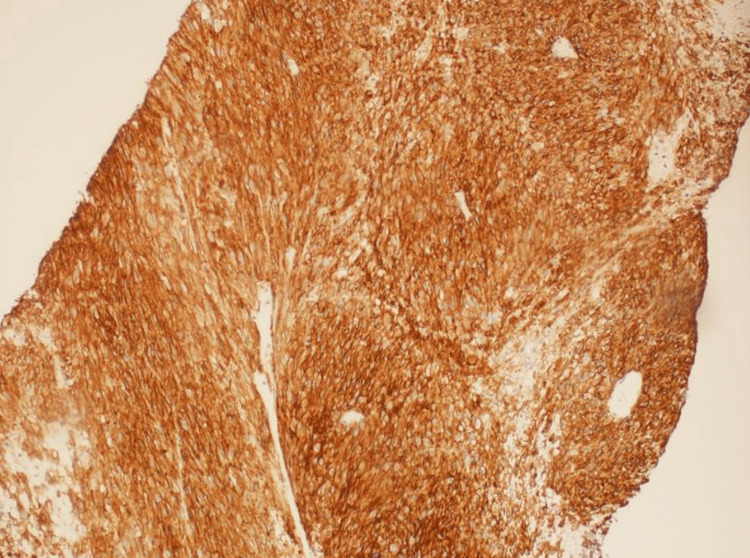
Immunostain of the Tru-Cut biopsy DOG1 immunostain showed diffuse strong positivity

**Figure 4:**
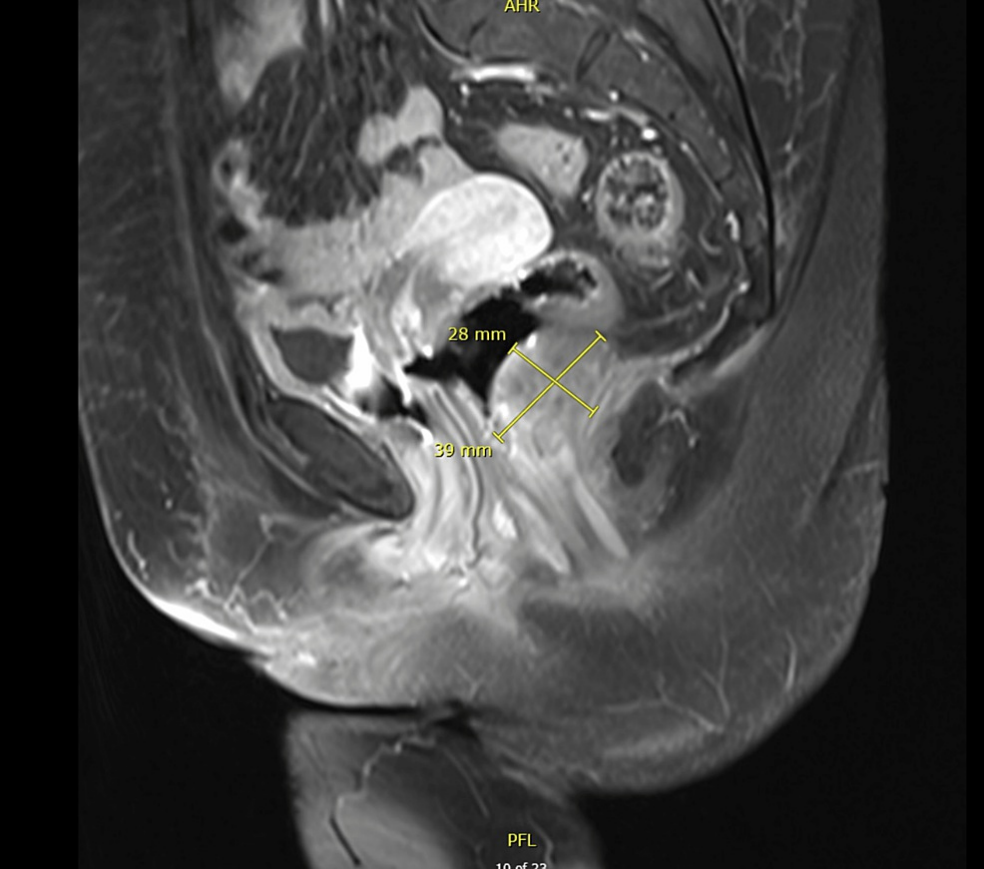
MRI post-neoadjuvant imatinib therapy MRI of the pelvis with saggital view showing the rectal tumor reduction in size to half post-neoadjuvant imatinib therapy

A 55-year-old female, with a past medical history of diabetes mellitus, presented to the emergency department with a five-month history of bleeding bright red blood per rectum, abdominal pain, and pain with defecation. The bleeding was very minimal with drops of blood in the stool that it was intermittent with no exacerbating factors. The abdominal pain was vague and continuous that lasted for two months with no radiation or exacerbating or relieving factors. Her symptoms were associated with all fatigue and generalized weakness but there was no loss of appetite or weight loss. On examination, she looked tired but oriented, no signs of anemia were detected, her abdomen was soft, and there were no detectable masses. A per-rectal examination revealed a rectal mass measuring 6 cm by 5 cm in the posterior rectal canal extending from 4 o’clock to 7 o’clock, the upper border was not palpable, and the lower border was 7 cm away from the anal verge. MRI of the pelvis was done and showed a mass measuring 6.3 cm by 5 cm, located 7 cm away from the anal verge as shown in Figure 1a and Figure 1b. CT scan was done and showed minimal regional peri rectal lymph node involvement. The patient had never had a colonoscopy done before; she also refused to undergo a colonoscopy at the time of diagnosis. She underwent an ultrasound-guided Tru-Cut biopsy that came to be a rectal gastrointestinal stromal tumor with a demonstration of spindle cell neoplasm and mitotic rate of more than 5 per 50 high power field, as shown in Figure 2a and Figure 2b. Immune stain showed diffuse positivity KIT +ve (Figure 3), in addition to DOG1 and CD34, SMA was locally positive, while Desmin and S100 both were negative. As the patient was also not willing for the surgery at the time of diagnosis, she was started on imatinib 400 mg/day. Following that, she experienced a hypersensitivity reaction to the given chemotherapy, as a result, the treating team decided to reduce the dose to 200 mg/day, which was well-tolerated by the patient. After three months, a repeat MRI pelvis was done and showed that the administration of imatinib led to a reduction of tumor size to 4.5 cm by 3.2 cm. A CT scan also showed a reduction in the size of the right internal iliac lymph node. The imatinib therapy was continued for another three months. A repeat MRI showed the tumor size to be 3.8 cm by 2.7 cm as shown in Figure 4. Finally, surgical resection was done. Since the tumor was located low in the rectum, only 7 cm away from the anal verge, the surgery was done completely through a direct trans-anal approach, there was no necessity to use any endoscopic techniques. In the lithotomy position, with the help of an anorectal retractor, the tumor was found posteriorly. It was resected with good free margins and muscles were approximated with interrupted sutures. The histopathology report postop showed the tumor size to be 3.5 cm by 2.2 cm, there was no capsular invasion, microscopic margins were tumor-free (R0), and there was no lymphovascular invasion. Pathological response to treatment revealed a reduction of the viable tumor to less than 10%. The postop pain was controlled with regular analgesia for three weeks. She was in a stable condition and most importantly, she had complete continence. After the surgical resection, she never experienced any fecal urgency, neither frequency nor loose stools. Lastly, to ensure complete resolution, she was resumed on adjuvant imatinib therapy after the surgery for a course of three years. A colonoscopy done after two years showed no recurrence. The patient has never experienced any postop complications and her overall quality of life is optimal.

## Discussion

GISTs are the most common mesenchymal tumors of the GIT. Cases were reported all over the GIT; the stomach accounted for the highest incidence, accounting for 51% of all GIST cases, followed by the intestine (36%); in comparison, rectal and colon GISTs are rare sites of GIST [1]. They were also identified in parts of the abdominal cavity like the omentum and mesentery [3]. Of all rectal tumors, GIST makes up 0.1% of all rectal masses [4]. The precursor cells of all the cases were from a common cell, which is the interstitial cell of Cajal in the mesenteric plexus, which is also the pacemaker cell of the tract. Cases were reported in all age groups, however, the median age of diagnosis is between 55 and 60 years. Clinical presentation varies according to the location of the tumor. In a review article, that was published in 2018 and included 35 rectal GIST cases, more than 50% of the presentation was latent, with no emergency or acute symptoms. Presentation is often gastrointestinal bleeding, intestinal obstruction, abdominal pain, or perforation. In fact, 30% of the cases were detected accidentally as part of a per-rectal examination in a gynecological assessment or during surgery or endoscopy [3]. In rare cases, urinary symptoms, such as urinary obstruction, were the first presentation [5].

There are three identifiable histological patterns, of which 70% are the spindle cell type, 20% are the epithelioid cell type, and the rest are a mixture of both [3]. GIST appears when there is a gain-of-function mutation in the c-KIT tyrosine kinase of the interstitial cells of Cajal, which makes tyrosine kinase continuously active [6]. KIT, also known as C117, is a tyrosine-protein kinase receptor type III, and it is found to be positive in 95% of GIST cases [7-8]. CD117 was not the only expressed marker, other markers include CD34 antigen, smooth muscle actin SMA, desman, and S100 protein [7]. In 2009, a retrospective review article of 29 patients showed the expression of CD34, SMA, and S-100 was found in, 28, 7, and 3 patients, respectively [6]. Markers vary according to the anatomical site of the tumor. The prognosis of the tumor depends on several factors, which most important are the tumor size, age, and mitosis index. Lymph node involvement doesn’t play a role, as lymphatic metastasis is very rare in rectal gist [9].

The mainstay of the management of localized rectal GIST is to excise the mass with microscopically negative margins. The type of surgical procedure performed depends on the size and location of the tumor. The options include trans-anal, trans-sacral, and trans-vaginal [9]. As for lower rectal tumors, which are small in size (up to 3 cm by 3 cm), with minimal extra rectal invasion, the preferred option would be the trans-anal approach. On the contrary, when the tumor size is exceeding 5 cm or there is a high element of extra rectal growth, the trans-rectal approach is not an option [10]. The introduction of imatinib chemotherapy as both neoadjuvant and adjuvant therapy has allowed the patients to become candidates for trans-rectal surgery, owing to its ability to result in several benefits, including decreasing the size of the tumor, decreasing mitotic activity, and decreasing recurrence. It also improves the quality of life by giving a chance to preserve the anus and prevent permanent colostomy. In an observational cohort study that was conducted on 19 patients with rectal GIST, nine of them underwent both neoadjuvant Imatinib and surgical resection of the tumor, and all of the nine patients were free from the disease [11].

The use of imatinib is accepted as adjuvant therapy for GISTs all over the world and is now the standard treatment for patients with intermediate to high risk of relapse [12]. In general, imatinib can be given to patients with GISTs, those who have a tumor size of more than 3 cm, and those who have a high mitotic rate, which is defined by the presence of more than five mitoses per 50 HPF. Those results were approved in a double-blinded placebo-controlled randomized trial that included patients with GIST KIT positive and at least 3 cm in diameter, it showed that imatinib significantly increased the recurrence-free survival over the placebo. On the other hand, when it comes to the mutational subtypes (KIT exon 11, exon 9 mutations, WT GIST), imatinib had no role in delaying the recurrence [13]. A systemic review that was published in August 2022 included patients with rectal GISTs all over the GI tract. The study concluded that receiving imatinib therapy followed by surgical resection had better survival than the patients who underwent surgical resection without preceding chemotherapy. However, the disease-free survival in both groups was the same [14].

## Conclusions

In establishing the differential diagnosis of a rectal mass, one should always keep in mind the possibility of a rectal GIST as a differential diagnosis. Although it’s a rare rectal cancer and may be kept last on the list, it is necessary to remember, as the treatment and management would differ from other rectal masses. CD117 and CD34 are important molecular markers that can help in the diagnosis of this rare tumor. Complete surgical resection is the definitive treatment, along with the rule of using imatinib as both neoadjuvant and adjuvant therapy, as it makes this goal achievable. However, further study is needed to understand the recurrence after imatinib therapy and the metastasis of the disease.
